# Targeted Metabolite Profiling-Based Identification of Antifungal 5-*n*-Alkylresorcinols Occurring in Different Cereals against *Fusarium oxysporum*

**DOI:** 10.3390/molecules24040770

**Published:** 2019-02-21

**Authors:** Ronald Marentes-Culma, Luisa L. Orduz-Díaz, Ericsson Coy-Barrera

**Affiliations:** Laboratorio de Química Bioorgánica, Universidad Militar Nueva Granada, Cajicá 250247, Colombia; u7500098@unimilitar.edu.co (R.M.-C.); luisa.orduz@unimilitar.edu.co (L.L.O.-D.)

**Keywords:** 5-*n*-alkylresorcinols, cereals, *Fusarium oxysporum*, microscale amended medium assay, supervised metabolite profiling

## Abstract

A rapid and convenient biochemometrics-based analysis of several cereal-derived extracts was used to identify *n*-alkyl(enyl)resorcinols (AR) as antifungals against *Fusarium oxysporum*. Total AR content and liquid chromatography/mass spectrometry (LC-MS)-based profiles were recorded for each extract, in addition to their antifungal activity, to help integrate these chemical and biological datasets by orthogonal partial least squares regression. In this study, we developed and used a micro-scale amended medium (MSAM) assay to evaluate the in vitro mycelial growth inhibition at low amounts of extracts. Triticale husk-derived extracts had the highest AR content (662.1 µg olivetol equivalent/g dry extract), exhibiting >79% inhibition at the highest doses (10.0–1.0 µg/µL). Correlation of the chemical and antifungal datasets using supervised metabolite profiling revealed that 5-*n*-nonadecanylresorcinol, 5-*n*-heneicosylresorcinol, and 5-*n*-tricosyl-resorcinol were the most active ARs occurring in cereal products from Colombia. Hence, we propose the biochemometrics-based approach as a useful tool for identifying AR-like antifungals against *F. oxysporum*.

## 1. Introduction

Cereals comprise some plant materials commonly called grasses—a merged name to designate those monocots of the Poaceae family. This family includes rice, wheat, rye, barley, oat, and other cereals, which represent the main source of food worldwide. Species within this family mostly produce a fruit called caryopsis (also grain or kernel). According to the Food and Agriculture Organization (FAO), 2610 million tons of cereals were produced in 2018, with wheat being the most prominent (754.1 million tons) [1]. In addition to their importance as a food supply, these grains are valuable materials for producing/accumulating several phytochemicals with potentially beneficial health effects, including reduced risk of diabetes, cancer, cardiovascular problems, chronic inflammation, neural degeneration, among other chronic disorders and diseases [2].

5-*n*-Alkyl(enyl)resorcinols (AR) are significant compounds found in cereals, which are specialized (or secondary) metabolites belonging to the phenolic lipids group, structurally composed by a *meta*-dihydroxy-substituted benzene ring and an alkyl (or alkenyl) chain at C-5 [3]. AR are commonly found in cereal grains, which so far have been their major source [3]. The highest AR contents have been reported in wheat bran, rye, and triticale, whereas maize, oats, and rice have smaller amounts [4,5]. ARs accumulate in the grain’s intermediate layers (hyaline layer, integument, and intermediate pericarp), and can be extracted via maceration or other emerging methodologies, such as extraction using supercritical CO_2_ and methanol or ethanol as co-solvents [6,7].

Several studies have demonstrated different nutritional and biological properties of ARs, such as antioxidant, anti-cancer, antifungal effects, etc. [8,9,10,11,12,13,14]. However, their antifungal activity against some phytopathogens has not been widely investigated. Some studies have reported on the activity against phytopathogens of alkylresorcinols obtained from rye [9,15,16,17], durum wheat [18], mango peel [19,20], and barley [21,22], which targeted phytopathogenic species from the genus *Fusarium*, *Penicillium*, and *Rhizoctonia* due to their ability to infect several crops. 

The genus *Fusarium* is a taxonomic group of filamentous, cosmopolitan fungi that can be both beneficial and harmful to plants, animals, and humans [23]. They are well-recognized pathogenic microorganisms that attack an extensive repertoire of plants and crops, causing damping-off, blotches, leaf boils, and dry rot in roots, stems, fruits, grains, and seeds [24]. In Colombia, four pathogenic species and 55 *Fusarium*-attacked plants have been recorded [25]. *Fusarium oxysporum* is the most important phytopathogen, which has been shown to infect >100 crop plants [26]. Several methods/strategies—biological and/or chemical-type controls—have therefore been attempted to control this species.

An alternative approach to control would be the use of naturally occurring compounds such as antifungal AR. However, the specific action or activity of individual and pure ARs remains unexplored due to isolation limitations associated with their chemical nature [22]. Therefore, the application of (un)targeted, supervised metabolomics for identifying a mixture’s active compounds has recently extended significance due to its advantages. This method integrates both biological and chemical datasets to determine which compounds of a mixture are responsible for the biological activity; an approach called biochemometrics [27]. In our current research on antifungals of plant origin, we evaluated several cereal-derived extracts for their *F. oxysporum* growth inhibition effects using a microscale amended medium assay (MSAM) and a mass spectrometry-based biochemometrics approach. Using this approach we integrated the antifungal activity and AR-based chemical composition, as a strategy to identify bioactive AR in a set of extracts of different cereals from Colombia. 

## 2. Results

### 2.1. Standardization and AR Quantification

Extraction from wheat bran using different solvents identified methanol as the best solvent for obtaining dry extracts (Table 1). On the other hand, acetone was the best solvent for extracting the highest AR amount (706.1 µg AR equivalent to olivetol per g of dry extract). Therefore, we chose acetone as a solvent for all other sample extractions. 

Within the analyzed samples, triticale husk (TH) extracts had the highest AR content, followed by wheat husk (WH) extracts, while the remaining samples showed intermediate contents (Table 2). In general, triticale and wheat yielded the highest, while rice, sorghum, and oats yielded the lowest AR contents.

### 2.2. Inhibition of Mycelial Growth on F. oxysporum

For the resultant 21 extracts, in vitro mycelial growth inhibition (MGI) against *F. oxysporum* was evaluated using the MSAM assay. Table 2 lists the MGI results for each extract and the corresponding doses. The data showed that the inhibition was dose-dependent, with values ranging 0.0–83.0% at 0.1–10.0 µg/µL doses, also exhibiting some significant differences between themselves (*p* < 0.05). Certificate oat husk (COH) samples showed the highest inhibition percentage, exceeding 80% in all three treatments. Triticale husk (TH) and triticale caryopsis (TC) also showed high inhibition percentages, while exhibiting a dose-dependent behavior. On the other hand, rice caryopsis (RC) samples had a lower inhibition percentage; a response only seen at the highest concentration (i.e., 10.0 µg/µL). 

### 2.3. Liquid Chromatography coupled to Electrospray Ionization Mass Spectrometry (LC-ESIMS) Analysis

**Figure 1:**
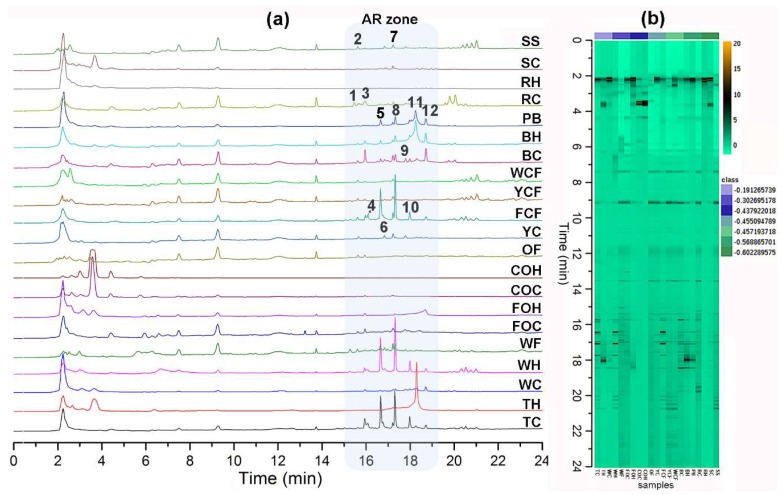
(**a**) Liquid Chromatography coupled to electrospray ionization mass spectrometry (LC-ESIMS) profiles; samples abbreviation according to Table 2; *n*-alkyl(enyl)resorcinols (AR) zone within chromatogram is highlighted in light blue box; bold numbers indicate the annotated AR according to Table 3; (**b**) LC-ESIMS data-derived heatmap using the Ward clustering algorithm and Euclidean distances; spots correspond to each detected compound by LC-ESIMS in the negative ion mode.

Positive ion mode did not give satisfactory LC-ESIMS profiles through electrospray ionization due to the phenolic nature of the ARs (data not shown). LC-ESIMS profiles (in negative ion mode) provided better information regarding composition differences between samples (Figure 1). A chromatographic method was particularly developed in this study for increasing AR’s separation procedure selectivity using reverse phase liquid chromatography. Due to the amphipathic nature of AR (very structurally related, differentiated by their side chains’ lengths and substitutions), this separation involved the addition of isopropanol (IPA) as a component of the organic modifier mixture (as eluent B, mixing IPA:MeOH (8:2)) as well as aqueous MeOH (as eluent A, mixing MeOH:H_2_O (8:2)), using gradient elution. This chromatographic method allowed the elution of AR compounds at 15–19 min (indicated as “AR zone”, Figure 1a), differentiated/separated from other polar and non-polar components, e.g., sugars, phenolics, organic acids, and triglycerides, among others (data not shown), which were also present in the extracts. A holistic view of all chromatograms is depicted in the respective heat map (Figure 1b), where the metabolic fingerprints exhibited remarkable differences in some samples but similarities in other zones between extracts. Triticale caryopsis-derived extracts exhibited the highest number of AR compounds. Putative identification at level three [28,29] is shown in Table 3. Twelve main AR-like compounds were then detected and identified (compounds **1**–**12**) [3,30]. 

### 2.4. Peak Annotation

In order to ensure a robust annotation and identification of AR-related metabolites, three mass spectrometry (MS)-based analyses were additionally performed on raw extracts: (1) high-resolution mass spectrometry using a quadrupole-time-of-flight (QToF) tandem mass analyzer and electrospray ionization (HRESIMS) in negative ion mode coupled to an Ultra-Fast Liquid Chromatography (UFLC) system, (2) low-resolution mass spectrometry using a single quadrupole mass analyzer and electrospray (LRESIMS) ionization in negative ion mode coupled to a UFLC system, and (3) electron-impact mass spectrometry (EIMS) using a quadrupole mass analyzer coupled to a gas chromatography (GC) system after derivatization using *N*-methyl-*N*-trimethylsilyltrifluoro-acetamide (MSTFA). Compilation of these data (Table A1) resulted in better peak annotation (Table 3). Thus, [M − H]^−^ and [2M − H]^−^ adducts that resulted from low-resolution (LR) and high-resolution (HR) ESIMS analyses, ensured a correct assignment through molecular ions and exact mass in agreement with the molecular formula for the identified metabolites (error < 4 ppm). Alkenylresorcinols such as **1**, **4**, **6**, and **11** exhibited ions in HRESIMS spectra, involving losses in the side chain by α-cleavage at double bonds (e.g., C_2_H_5_OH, C_2_H_2_, and C_10_H_16_). The resorcinol moiety of compounds **1**–**12** was also established after derivatization using *N*-methyl-*N*-trimethylsilyl-trifluoroacetamide activated II^®^ (MSTFA), following the protocol previously described [31]. All compounds shared very similar fragmentation patterns to silylated AR, indicating the homologous nature of all identified compounds [3]. Thus, the resulting trimethylsilyl (TMS) ether derivatives were analyzed using GC/EIMS, and the characteristic fragment at *m*/*z* 268 (base peak) was a distinctive signal for the di-*O*-silylated 1,3-dihydroxytropylium cation ([(TMSO)_2_C_6_H_4_CH_2_]^+^), as well as fragments at *m*/*z* 281, 310, and 341, which are very common for ARs [32,33]. According to the quasi-molecular ions recorded in LR and HRESIMS spectra, the respective molecular ion for each di-*O*-silylated derivative of compounds **1**–**12** was consequently detected ([M + (TMS)_2_]^+^). Differences between the fragment at *m*/*z* 268 and molecular ion, indicated the particular length for the side chain, which in turn involved consecutive losses of CH_2_ fragments [30,31].

### 2.5. Multivariate Analysis

**Figure 2:**
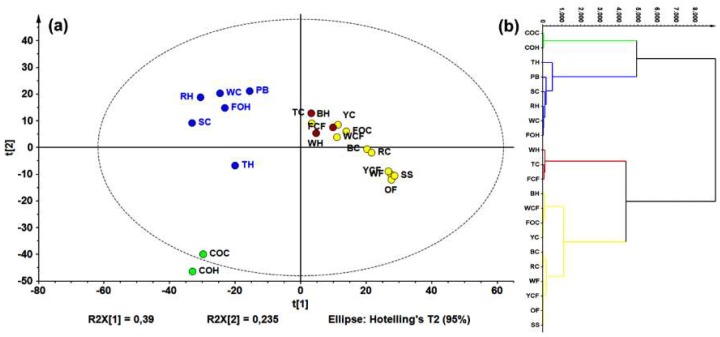
Principal component analysis (PCA) on liquid chromatography-mass spectrometry (LC-MS) data for all samples. (**a**) Principal component 1 (PC1) vs. PC2 score plot (R^2^_Xcum_ = 0.625), colors according to hierarchical clustering analysis (HCA); and (**b**) HCA-derived dendrogram over PCA data.

LC-HRESIMS data (in negative mode) were examined using principal components analysis (PCA, Figure 2a). The resulting model explained that 80% of the data’s total variance was from the first three components. Using a hierarchical clustering analysis (HCA) on PCA data points (Figure 2b), four groups were identified.

**Figure 3:**
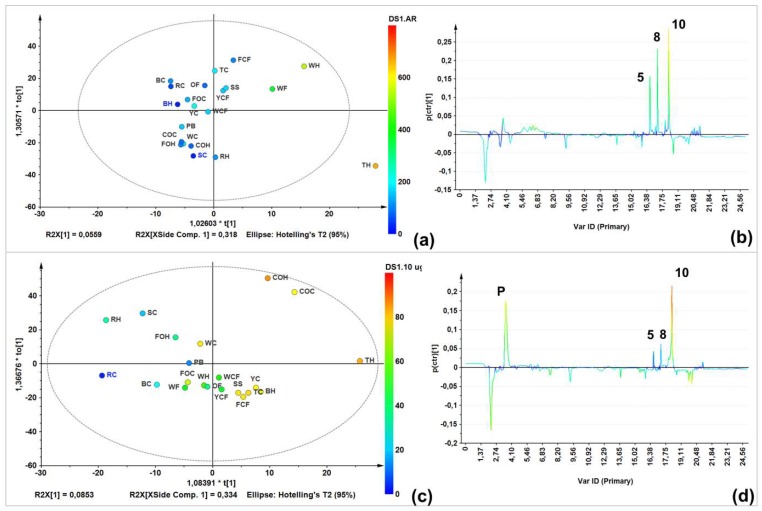
Targeted analysis using supervised statistics (biochemometrics). (**a**) Single-Y orthogonal partial least-squares regression (OPLS)-derived score plot on LC-MS (in negative ion mode) data using total AR content (TARC) as supervision variable; (**b**) *S*-line projection on LC-MS (in negative ion mode) data and TARC values; (**c**) Single-Y OPLS-derived score plot on LC-MS (in negative ion mode) data using antifungal activity at 10 µg/µL as supervision variable; and (**d**) *S*-line projection on LC-MS (in negative ion mode) data and antifungal activity at 10 µg/µL.

A single-Y orthogonal partial least squares (OLPS) analysis was therefore performed to visualize the relationship between LC-MS profiles and total AR contents. The score plot (Figure 3a) showed a good AR content-related distribution. Furthermore, the *S*-line projection (Figure 3b) was then constructed to determine those variables/signals obtained in the LC-ESIMS analysis (in negative mode) and their relation to the AR total content. Three signals were correlated to AR-rich samples eluted at 15–19 min. Similarly, another OPLS model was built to integrate LC-ESIMS profiles and inhibition data, to determine the plausible relationship between the chemical composition and antifungal activity (Figure 3c). Inhibition at a higher dose (10 µg/µL) was used because all samples inhibited mycelial growth at this dose to different extents, thus incorporating a better distribution depending on growth inhibition. Respective *S*-line projection identified three activity-correlating signals (Figure 3d).

## 3. Discussion

Among the solvents used for AR extraction, acetone extracted the highest amount of target compounds compared to the other three solvents according to Tukey’s test (*p* < 0.05). Although there is no statistical significance compared to methanol, acetone was chosen due to its nature—intermediate-to-high polarity, which matches the amphipathic characteristic of ARs—and ability to extract more diverse ARs [30]. Acetone also has low environmental impact, and is therefore more easily removed from the extract matrix [34,35].

Colorimetric AR quantification indicated that all extracts contain these metabolites at different levels (i.e., 24–660 µg olivetol equivalent (OE)/g dry extract (DE) range), although some have been reported to be absent [36]. The highest AR content was found in triticale husk (TH) (662.1 µg OE/g DE), followed by wheat husk (WH) (541.7 µg OE/g DE). While in kernels, the triticale caryopsis (TC) (185.4 µg OE/g DE) and wheat caryopsis (WH) (177.5 µg OE/g DE) had higher AR contents (Table 2). These metabolites have been found to accumulate at higher levels in different layers of the caryopsis, such as the hyaline layer, testa, and inner pericarp [6], while there is no record of them existing in husk. Consistently, Athukorala et al. [7] reported higher AR contents in a triticale (a wheat-rye hybrid) cultivar (700 µg/g dry bran) compared to wheat, with the highest content recorded in bran rye (750 µg/g dry bran). Lower AR contents were found in other rye products [37]. In addition, triticale caryopsis had the highest variety of these metabolites, according to the negative-mode LC-ESIMS analysis. Compounds such as C_17_, C_19_, C_21_, C_23_, and C_25_-type alkylresorcinols were then recorded [38,39,40]. In the present study, cultivated soybean seeds (SS; used for human consumption) also exhibited AR presence. Such compounds are not exclusive to the Poaceae species, since some Fabaceae plants contain AR, which has been reported in *Ononis* genus [41].

Regarding unsupervised analysis on LC-ESIMS data, four clusters were found according to HCA (Figure 2b), which are shown in a two-component plots (Figure 2a). Within the group possessing the highest variance compared to the others (green), there was a group involving commercial oats samples (caryopsis and husk); a second (red) comprised rice caryopsis, barley, wheat flour, and oat flakes; the blue group contained samples with weak inter-relations such as triticale husk, forage oats, and rice; and the yellow group involved triticale and forage oat caryopsis. Wheat flour had a lower AR content compared to the other samples (e.g., husk and caryopsis wheat), this is mainly caused by the fact that during refining, grinding, and sieving processes, too much of the pericarp and husk is eliminated. Similar condition can occur with corn flour, which can explain these samples clustering in different groups compared to other extracts from the same species. This statistically-based comparison of chemical compositions between extracts can rationalize the observed activity against *F. oxysporum*. In fact, a comparison between the LC-ESIMS profiles and total AR contents (through construction of a single-Y OLPS) indicated a composition-related distribution associated with their AR content. Thus, samples with the lowest AR content were in the third quadrant, while those with the highest content clustered in the first quadrant. The respective *S*-line projection revealed three features (i.e., LC-ESIMS signals) that correlated with total AR contents of all extracts. These features are then related to compounds **5**, **8**, **10** (Figure 3b).

In the context of evaluating extracts against *F. oxysporum* using the MSAM method, MGI could be assessed even when extract amounts are low, which is a particular limitation during screening initiatives as well as for validation purposes, due to the lack of replicates and a suitable method for evaluating MGI at a microscale level. The MSAM protocol (using low amounts of medium and inputs) produced reproducible results for the activity, generating a smaller quantity of waste, which is the common problem in the antifungal-amended medium protocol in petri dishes.

Some experimental studies have indicated that plants synthesize ARs to create a chemical barrier for resisting fungal attacks [42,43,44]. Therefore, the antifungal activity of these products is attributed to the presence of ARs. According to MSAM assays against *F. oxysporum*, the highest activity was found for certificate oat husk (COH), which exhibited >80% inhibition in all three doses. However, these samples were purchased with commercial certification for starting a crop (no human/animal consumption). These materials are treated with different commercial antifungals [45], which guarantee caryopsis protection to various post-harvest products that affect pathogens such as *Fusarium*, *Rhizoctonia*, *Penicillium*, and *Aspergillus* [15,17]. In fact, this certified oat caryopsis and husk were previously treated with phenylpyrrole fludioxonil, which we used as an internal standard. Due to the above fact, the high inhibition was therefore attributed to this agent being extracted by acetone and being active within the extract. The LC-ESIMS profile of COH prominently differed from other samples (even from other oat samples), which was evident from the unsupervised analysis by its higher variance (Figure 2a). Caution is therefore advised when polluted botanical extracts are evaluated in order to avoid false positives. 

The results in Table 2 agree with those previously published [9], where a mixture of C_13_-C_27_ saturated 5-*n*-alkylresorcinols was tested against three phytopathogens (*F. culmorum* (WG Sm.) Sacc., *Rhizoctonia solani* JG Kühn, and *R. cerealis* EP Hoeven). The authors found that this mixture was inhibitory at different levels; phytopathogens were inhibited at 10–20 µg/µL, with *F. culmorun* being the most treatment resistant. When comparing our present results, we found more bioactivity in some test extracts, where the highest growth inhibition was observed at 1.0 µg/µL, whereas previous studies achieved similar inhibition at 5–10 µg/µL [9]. Triticale (husk and caryopsis) exhibited the highest inhibitory effect at 10.0 and 1.0 µg/µL (75–83% inhibition range). Triticale husk had the highest AR concentration (Table 2), whereas triticale caryopsis did not present a high AR content compared to other samples, however, it contained the highest number of identified compounds. From the MS-based identification, a single compound with several unsaturations was found in triticale (identified as 5-*n*-heptacosatetraenylresorcinol, C_27:4_), which has also been reported in rye, the triticale progenitor [3]. 

In this context, information regarding extract composition and antifungal activity separately did not reveal an association with presence/absence of bioactive ARs. Therefore, integrating the mycelial growth inhibition (dependent) with LC-ESIMS (independent) data as a tool for identifying antifungal components, the subsequent biochemometrics-based analysis through the internal cross-validated construction of a single-*Y* OPLS model (Figure 3c) evidenced some correlation based on bioactivity (R^2^_Xcum_ = 0.719). The respective *S*-line displayed graphically the influence and correlation of loadings against the dependent variable. For this biochemometrics-based model, the upper quadrant of the *S*-line contributed the most to biologically differentiating more versus less active extracts. Thus, 5-*n*-nonadecanylresorcinol (**5**), 5-*n*-henicosylresorcinol (**8**), and 5-*n*-tricosylresorcinol (**10**) were identified as possessing the highest contribution to the observed high mycelial growth inhibition. Compound **10** exhibited the highest correlation value in *S*-line (ca. 80%) (Figure 3b). These identified chemical features are in agreement with a previous study describing that saturated and longer-chain AR may retain antifungal activity for longer, since some phytopathogenic fungi have the capacity to metabolize relatively shorter chain alkylresorcinols, transforming them into less toxic compounds [15]. These compounds can be consequently considered as promising antifungals for inclusion in further studies to validate these findings. Other phenolics (signals within 2–14 min) exhibit no correlation with antifungal activity. In fact, the abundant compound at 2.2 min (ferulic acid, *m*/*z* 193 [M − H]^−^) was present in samples with lower activity, indicating no relationship with antifungal action. In contrast, a compound marked as P (3.7 min, Figure 3d) was identified as the phenylpyrrole fludioxonil (*m*/*z* 247 [M − H]^−^), the fungicidal ingredient used to protect the certified oat caryopsis. This reference material with this fungicide were used as internal standards and, as observed in the respective *S*-line (Figure 3d), this compound correlated with antifungal activity but not AR contents. These facts therefore confirm the usefulness of this biochemometric model for AR-like antifungals identification.

## 4. Material and Methods

### 4.1. Fungal and Plant Material

Plant materials of some cereals were obtained from several places in Colombia. Caryopsis and husk of barley, triticale, yellow corn, and wheat were gathered from an experimental crop at Nueva Granada Campus in Cajicá, Colombia; caryopsis and husk of rice, oat, pearl barley, soy, and sorghum, wheat flour, corn (forage, white and yellow) flour, and oat flakes were purchased from different marketplaces in Bogotá, Colombia. All aforementioned materials were ensured to be free of pesticide traces based on the organic cultivation protocol implemented for human/animal consumption. In addition, certificate oat material (husk and caryopsis), previously treated with fludioxonil, was also purchased from a marketplace in Bogotá, Colombia for use as reference material. The *Fusarium oxysporum* strain was obtained from a Cape gooseberry (*Physalis peruviana*) crop, preserved on Whatman paper # 1 at −20 °C, and reactivated in Potato Dextrose Agar (PDA) at full concentration before use.

### 4.2. Extracts Standardization and Total AR Quantification

All kernel samples were grinded in a Waring WGS30 grinder machine (Waring Commercial, Stamford, CT, USA), and the husk was separated from the flour using a stainless steel sifter (18 cm diameter, mesh 40) prior to the extraction. The best AR extraction solvent was determined as follows: a 24-h maceration extraction was carried out on wheat bran (1 g) with different solvents (10 mL; *n*-hexane, ethyl acetate, isopropanol, acetone, and methanol). The resulting mixture was then filtered and concentrated under reduced pressure to obtain the unfractionated, crude extract in each solvent. 

Total AR content (TARC) was determined by the colorimetric quantification method reported by Sampietro et al. [46], using the Diazonium salt Fast Blue^®^ RR (FBRR^®^) with some modifications. Briefly, a working solution was prepared at 1:5 ratio with methanol (stock 0.5 mg/mL FBRR^®^), and a solution to 5 mg/mL of the samples previously prepared. An aliquot of extract solution (20 µL) was added, FBRR^®^ working solution (2 mL) and 10% sodium carbonate (Na_2_CO_3_; 10 µL) were dispensed into spectrophotometric cells. These cells were incubated for 20 min at room temperature and the absorbance was measured on a Genesys 20 spectrophotometer (Thermo Scientific, Waltham, MA, USA) at 480 nm. Total AR content was then calculated from a previously constructed calibration curve using olivetol (5-pentylresorcinol) as standard. The selected solvent (i.e., acetone) was allowed to remove AR from other samples using 60 g of plant material for the respective extract preparation. Thus, the procedure described previously for wheat bran was therefore performed for all samples. TARC values were expressed as µg olivetol equivalent per g dried extract (µg OE/g DE).

### 4.3. Extraction and Chemical Analyses for Metabolite Profiling

#### 4.3.1. Preparation of AR-Rich Extracts from Different Cereals

Once the extraction procedure was standardized/optimized as described in Section 4.2, 21 different dry, ground cereal materials were used for chemical analyses. A 24-h maceration extraction using acetone (10 mL) was performed on cereal material (1 g). After extraction, the resulting mixture was filtered and concentrated under reduced pressure to obtain the unfractionated, crude AR-rich extracts. All extracts were stored at −20 °C until respective use in chemical analyses and antifungal assay.

#### 4.3.2. High Performance Liquid Chromatography-Electrospray Ionization-Mass Spectrometry (LC-ESI-MS) Analysis

All extracts were analyzed using Reverse Phase Ultra-Fast Liquid Chromatography coupled to a microTOFQ II mass spectrometer (Bruker, Billerica, MA, USA). A Kinetex^®^ column (150 × 4.6 mm, 2.6 µm) was used for analysis at 0.6 mL/min using the mixtures MeOH:H_2_O (8:2) (A) and isopropanol:MeOH (3:7) (B) in a gradient method (0–2 min 0% B, 14–23 min 100% B, and 25–30 min 0% B). Electrospray ionization interface (ESI) was operated in a negative ion mode (scan 100–2000 *m*/*z*), desolvation line temperature at 250 °C, nitrogen as nebulizer gas at 1.5 L/min, drying gas at 8 L/min, quadrupole energy at 7.0 eV, and collision energy 14 eV.

Extracts were also analyzed by Reverse Phase Ultra-Fast Liquid Chromatography coupled to a Diode Array Detector (UFLC-DAD) on a Prominence system (Shimadzu, Columbia, MD, USA), coupled to a Shimadzu LC2020 mass spectrometerA Kinetex^®^ column (150 × 4.6 mm, 2.6 µm) was used for analysis at 0.6 mL/min using the mixtures MeOH:H_2_O (8:2) (A) and isopropanol:MeOH (3:7) (B) in a gradient method (0–2 min 0% B, 14–23 min 100% B, and 25–30 min 0% B); detection was performed at 270 nm. Electrospray ionization interface (ESI) was operated simultaneously in positive and negative ion modes (scan 100–2000 *m*/*z*), desolvation line temperature at 250 °C, nitrogen as nebulizer gas at 1.5 L/min, drying gas at 8 L/min, and detector voltage at 1.4 kV.

#### 4.3.3. Gas Chromatography-Electron Impact Mass Spectrometry (GC/EIMS) Analysis

Raw extracts were derivatized with *N*-methyl-*N*-trimethylsilyltrifluoroacetamide activated II^®^ (Sigma, St. Louis, MO, USA). For each sample (5 mg/mL), 200 µL were dried into a chromatography vial with and insert; 20 µL of the derivatization agent were then added and incubated at 60 °C for one hour [31]. The analysis was carried out using a Trace 1300 LT gas chromatograph (Thermo Scientific, Waltham, MA, USA) equipped with an AI 1310 auto sampler, coupled to a Thermo ISQ Mass Spectrometer (using electron impact ionization and a single quadrupole analyzer), with a 60 m × 0.25 mm ID × 0.25 µm DB-5 column (Rxi^®^ 5 Sil MS, Restek, State College, PA, USA) using helium (99.999%), with a flow of 1.5 mL/min, 1 µL of sample was injected in splitless mode, and the temperature of the injection port was 300 °C. The temperature program started with an initial temperature of 100 °C maintained for 1 min, then increasing from 100 °C to 250 °C at 15 °C/min, and holding for 5 min. It was then increased to 310 °C at 10 °C/min, and maintained for 30 min. The ion source and transference line temperatures were 250 °C and 310 °C, respectively.

### 4.4. Microscale Amended Medium (MSAM) Assay

**Figure 4:**
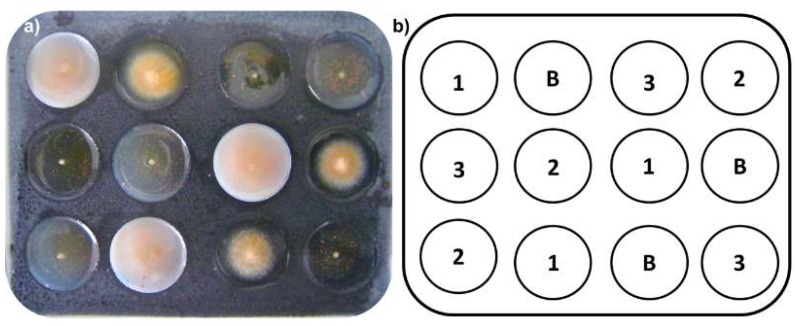
(**a**) Experimental unit (glass 12-well plates) for micro-scale amended medium (MSAM) assay. Each well was filled with medium (150 μL) previously amended with extracts and then inoculated (*F. oxysporum*) using 1-mm plugs placed onto the center of each well. (**b**) A schematic representation of the randomly-organized experimental design for the MSAM assays; B = blank (*F. oxysporum* without treatment), each number (1, 2, and 3) indicates the treatment (in µg/µL) loaded into each well of the 12-well plate: 1 = 10 µg/µL; 2 = 1.0 µg/µL; 3 = 0.1 µg/µL.

In vitro evaluation of mycelial growth inhibition against *F. oxysporum* was performed using an innovative, micro scale amended protocol using 12-well glass plates. A previously stirred mixture (150 µL) containing Potato Dextrose Broth PDB (25.0 g/L) and bacteriological agar (0.5%) amended with extracts (distinctly adding the required amount of extract for succeeding the desired final concentration, such as 10.0, 1.0 and 0.1 µg/µL, as treatment) was placed into each well as culture medium. The required amount of extracts to reach the final concentration was added to the warm medium, and the mixture was vigorously stirred using a vortex for 3 min until adequate dispersion was achieved, which resulted in the amended media. A 1-mm agar-mycelial plug from actively growing *F. oxysporum* cultures was inoculated onto the center of each well. Each test consisted of a randomized design with three replicates compared to a plate control (untreated medium; Figure 4). Prochloraz^®^ was used as positive control. Each 12-well plate was placed into a humid chamber and allowed to incubate for 72 h at room temperature (average, 17 °C).

The plates were then photographed in a stereoscope (Optika^®^ SZM-LED2, Ponteranica, Italy) and analyzed using ImageJ software (bundled with 64-bit Java 1.8.0_112, NIH, Bethesda, MD, USA, https://imagej.net). The inhibition percentage was calculated based on the area measured by ImageJ, compared to the control area, according to Equation (1):

(1) $$\%\;inhibition=100-\left(\frac{Sample\;area}{Control\;area}\;\times \;100\right)$$

### 4.5. Statistical Analyses

Total contents and antifungal activity data were subjected to the Shapiro–Wilks test, parametric analysis of variance (ANOVA), and Tukey’s test (*p* < 0.05) using R project software version 3.0.2 (R Foundation, Vienna, Austria). In addition, the AR contents, ASCII-formatted LC-MS-derived data (previously aligned, normalized and autoscaled), and growth inhibition percentages against *F. oxysporum* were used to build the dataset. The resulting matrix was used for multivariate statistics (biochemometrics) through Single-Y orthogonal partial least squares (OPLS) regression using the SIMCA 13.0 software (Umetrics Inc., Umeå, Sweden).

## 5. Conclusions

AR-rich extracts from cereals showed inhibitory activity against *F. oxysporum* at different levels depending on the particular AR present. In this sense, negative ion mode LC-ESIMS profiles of acetone extracts revealed the presence of at least twelve main ARs. These findings were then supported by biochemometrics analysis combined with the development of a micro-scale amended medium (MSAM) protocol, which allowed us to evaluate small amounts of cereals-derived extracts. This combination (i.e., biochemometrics and MSAM) led to the identification of bioactive AR without the need for multiple bioactivity-guided isolation steps, thus improving the efficiency and productivity of antifungal discovery purposes. Therefore, multivariate statistical modeling (based on single-Y OPLS) revealed three ARs from a set of extracts that were responsible for the observed mycelial growth inhibition such as compounds **5**, **8**, and **10**, which were also correlated by OPLS to the AR-rich mixtures. The results therefore indicate that these compounds, abundant in cereals, are important and valuable chemical entities that could eventually be included into advance experiments focused on *F. oxysporum* control. Further studies (e.g., biochemometrics-guided fractionation) are also required for isolating these compounds and to further validate our findings.

## Figures and Tables

**Table 1:** *n*-Alkyl(enyl)resorcinol (AR) extraction using different organic solvents.

Solvent	Extraction Yield (%*w*/*w*)	Total AR Content ^a^ (µg OE/g DE)
Acetone	3.9 ± 0.2 ^B^	706.1 ± 23.2 ^A^
Methanol	6.2 ± 0.4 ^A^	679.6 ± 32.1 ^AB^
*n*-Hexane	4.3 ± 0.3 ^B^	650.5 ± 21.3 ^B^
Ethyl acetate	3.1 ± 0.2 ^D^	510.7 ± 35.5 ^C^
*iso*-Propanol	3.5 ± 0.1 ^C^	497.2 ± 28.6 ^C^

^a^ Total AR Content in µg olivetol equivalent/g dry extract (µg OE/g DE). All values are expressed as mean ± standard deviation using data from three replicates. Different superscript capital letters reflect statistically significant differences between samples according to Tukey’s test.

**Table 2:** AR quantification for each processed sample, growth inhibition for each evaluated sample at three different concentrations, and their statistical classification.

Samples	Code	CO ^a^	EY ^b^	TARC ^c^	MGI ^d^ (%)		
					10.0 µg/µL	1.0 µg/µL	0.1 µg/µL
Barley Husk	BH	Co	0.12	24.7 ± 0.5 ^M^	74.8 ± 1.5 ^AB^	70.6 ± 1.8 ^BC^	12.9 ± 1.8 ^BCD^
Forage Oat Husk	FOH	Cu	0.693	104.8 ± 3.3 ^IJ^	36.4 ± 11.3 ^EF^	17.8 ± 1.6 ^FG^	19.1 ± 5.6 ^BC^
Rice Husk	RH	Co	0.509	115.8 ± 4.2 ^I^	33.1 ± 1.7 ^EFG^	16.2 ± 6.2 ^FG^	11.7 ± 1.4 ^CD^
Triticale Husk	TH	Cu	1.659	662.1 ± 20.4 ^A^	82.5 ± 0.3 ^AB^	79.4 ± 1.6 ^AB^	18.8 ± 3.0 ^BC^
Wheat Husk	WH	Cu	1.734	541.9 ± 24.7 ^B^	56.6 ± 2.6 ^CD^	21.3 ± 0.2 ^DEF^	26.5 ± 1.2 ^B^
Pearl Barley	PB	Co	0.666	156.3 ± 2.4 ^GH^	13.5 ± 2.3 ^HI^	11.5 ± 0.4 ^G^	7.5 ± 7.7 ^CD^
Certificate Oat Husk	COH	Co	0.451	81.8 ± 3.7 ^K^	85.6 ± 0.2 ^A^	80.7 ± 0.2 ^A^	80.7 ± 1.4 ^A^
Certificate Oat Caryopsis	COC	Co	0.596	86.2 ± 3.0 ^JK^	75.1 ± 2.9 ^AB^	65.5 ± 0.1 ^C^	19.2 ± 1.2 ^BC^
Forage Corn Flour	FCF	Co	0.724	103.9 ± 4.4 ^IJ^	47.7 ± 2.7 ^DE^	19.9 ± 0.8 ^EFG^	9.9 ± 1.1 ^CD^
Oats Flakes	OF	Co	0.591	85.1 ± 5.2 ^JK^	36.5 ± 5.7 ^EF^	23.0 ± 1.2 ^DEF^	8.2 ± 0.7 ^CD^
Barley Caryopsis	BC	Co	0.648	117.5 ± 2.0 ^I^	25.0 ± 3.2 ^FGH^	18.9 ± 1.0 ^EFG^	11.6 ± 0.5 ^CD^
Forage Oat Caryopsis	FOC	Cu	0.796	139.2 ± 2.5 ^H^	69.9 ± 0.6 ^BC^	16.5 ± 3.0 ^FG^	11.7 ± 0.1 ^CD^
Rice Caryopsis	RC	Co	0.251	56.6 ± 1.4 ^L^	3.9 ± 0.4 ^I^	0.0 ± 0.0 ^H^	0.0 ± 0.0 ^D^
Sorghum Caryopsis	SC	Co	0.146	26.2 ± 0.7 ^M^	19.5 ± 0.3 ^GH^	16.2 ± 0.8 ^FG^	10.7 ± 6.6 ^CD^
Soy Seed	SS	Cu	1.039	168.2 ± 2.0 ^EFG^	77.4 ± 0.7 ^AB^	25.2 ± 0.2 ^DEF^	13.7 ± 1.7 ^BCD^
Triticale Caryopsis	TC	Cu	0.648	185.4 ± 6.9 ^E^	78.5 ± 3.7 ^AB^	75.7 ± 4.7 ^AB^	18.4 ± 2.7 ^BC^
Wheat Caryopsis	WC	Cu	0.688	177.5 ± 4.6 ^EF^	76.9 ± 1.3 ^AB^	63.4 ± 2.1 ^C^	11.9 ± 3.9 ^CD^
Wheat flour	WF	Co	1.314	363.0 ± 16.8 ^C^	77.2 ± 4.3 ^AB^	30.1 ± 4.0 ^D^	8.4 ± 0.1 ^CD^
White Corn Flour	WCF	Co	1.131	162.8 ± 4.6 ^FG^	52.0 ± 2.1 ^D^	15.9 ± 0.3 ^FG^	7.4 ± 5.5 ^CD^
Yellow Corn	YC	Cu	1.131	215.7 ± 3.2 ^D^	75.2 ± 0.7 ^AB^	27.6 ± 1.9 ^DE^	15.5 ± 0.5 ^BC^
Yellow Corn Flour	YCF	Co	1.093	157.7 ± 5.6 ^FGH^	46.0 ± 5.6 ^DE^	23.1 ± 2.1 ^DEF^	18.6 ± 6.3 ^BC^

^a^ Cereal origin (CO): Co = commercially purchased in Bogotá, Colombia, Cu = cultivated in the Nueva Granada campus, Cajicá, Colombia; ^b^ Extraction Yield (EY) in %*w*/*w*; ^c^ Total 5-*n*-alk(en)ylresorcinol (AR) Content (TARC) in µg Olivetol equivalent/g dry extract (DE); ^d^ mycelial growth inhibition (MGI); data expressed as mean ± standard deviation using three replicates. Different superscript capital letters reflect statistically significant differences between samples according to Tukey’s test.

**Table 3:** Annotated 5-*n*-alk(en)ylresorcinols homologues in acetone extracts, using LC-ESIMS (in negative ion mode).

No	Rt (min) ^a^	[M − H]^−^ (*m*/*z*) ^b^	Error (ppm)	Molecular Formula ^c^	Homologue	Name
**1**	15.4	389.3048	−1.80	C_25_H_42_O_3_	C19:1, OH	5-*n*-(hydroxynonadecenyl)resorcinol
**2**	15.6	455.3896	1.54	C_31_H_52_O_2_	C25:2	5-*n*-(pentacosadienyl)-resorcinol
**3**	15.9	347.2956	1.73	C_23_H_40_O_2_	C17	5-*n*-heptadecylresorcinol
**4**	16.1	373.3113	1.88	C_25_H_42_O_2_	C19:1	5-*n*-nonadecenylresorcinol
**5**	16.7	375.3269	1.60	C_25_H_44_O_2_	C19	5-*n*-nonadecanylresorcinol
**6**	16.9	401.3413	−1.49	C_27_H_46_O_2_	C21:1	5-*n*-henicosenylresorcinol
**7**	17.2	445.3693	2.69	C_29_H_50_O_3_	C23, Oxo	5-*n*-(oxotricosanyl)-resorcinol
**8**	17.4	403.3586	2.48	C_27_H_48_O_2_	C21	5-*n*-henicosylresorcinol
**9**	17.7	415.3201	−2.65	C_27_H_44_O_3_	C21:1, Oxo	5-*n*-(oxoheneicosenyl)-resorcinol
**10**	18.0	431.3903	3.25	C_29_H_52_O_2_	C23	5-*n*-tricosylresorcinol
**11**	18.3	479.3899	2.09	C_33_H_52_O_2_	C27:4	5-*n*-heptacosatetraenylresorcinol
**12**	18.8	459.4216	3.05	C_31_H_56_O_2_	C25	5-*n*-pentacosylresorcinol

^a^ R_t_ = retention time (in min); ^b^ high-resolution mass spectrometry using a quadrupole-time-of-flight (QToF) tandem mass analyzer and electrospray ionization in negative ion mode coupled to an Ultra-Fast Liquid Chromatography (UFLC) system; ^c^ molecular formula deduced from analyses of mass spectra data (see Table A1).

## Appendix A

**Table A1:** MS-based analyses of compounds **1**–**12** using three MS detectors coupled to chromatography: LC/LRESIMS LC/HRESIMS, GC/EIMS (after MSTFA derivatization).

Comp.	HRESIMS ^a^	LRESIMS ^b^	EIMS ^c^
	*m*/*z* (%), Cluster	*m*/*z* (%), Cluster	*m*/*z* (%), Fragment
**1**	389.3028 (85) [M − H]^−^	389 (100)	268 (100) [(TMSO)_2_C_6_H_4_CH_2_]^+^ 534 (10) [M + (TMS)_2_]^+^
	375.3259 (100), [M − CH_2_-H]^−^		
	343.3020 (24), [M − C_2_H_5_OH]^−^		
**2**	455.3891 (15) [M − H]^−^	455(70), [M − H]^−^	268 (100) [(TMSO)_2_C_6_H_4_CH_2_]^+^ 600 (10) [M + (TMS)_2_]^+^
		296 (60)	
		574(50)	
**3**	347.2976 (100), [M − H]^−^	347 (100), [M − H]^−^	268 (100) [(TMSO)_2_C_6_H_4_CH_2_]^+^ 492 (30) [M + (TMS)_2_]^+^
	695.5997 (6), [2M − H]^−^	695 (60), [2M − H]^−^	
**4**	373.3130 (100), [M − H]^−^	373 (100), [M − H]^−^	268 (100) [(TMSO)_2_C_6_H_4_CH_2_]^+^ 518 (20) [M + (TMS)_2_]^+^
	347.2971 (30), [M − C_2_H_2_-H]^−^	747 (30), [2M − H]^−^	
	747.6278 (5), [2M − H]^−^	398 (10)	
**5**	375.3289 (100), [M − H]^−^	375 (100), [M − H]^−^	268 (100) [(TMSO)_2_C_6_H_4_CH_2_]^+^ 520 (40) [M + (TMS)_2_]^+^
	751.6652 (13), [2M − H]^−^	751 (40), [2M − H]^−^	
**6**	401.3413 (40), [M − H]^−^	401 (100), [M − H]^−^ 803 (15), [2M − H]^−^	268 (100) [(TMSO)_2_C_6_H_4_CH_2_]^+^ 546 (10) [M + (TMS)_2_]^+^
	375.3279 (100), [M − C_2_H_2_-H]^−^		
	803.6830 (5), [2M − H]^−^		
**7**	446.3693 (50) [M − H]^−^	446 (50), [M − H]^−^	268 (80) [(TMSO)_2_C_6_H_4_CH_2_]^+^ 591 (30) [M + (TMS)_2_]^+^
		1190 (60)	
**8**	403.3596 (100), [M − H]^−^	403 (100), [M − H]^−^	268 (100) [(TMSO)_2_C_6_H_4_CH_2_]^+^ 548 (30) [M + (TMS)_2_]^+^
	807.7275 (45), [2M − H]^−^	807 (45), [2M − H]^−^	
**9**	415.3187 (80) [M − H]^−^	415 (65), [M − H]^−^	268 (100) [(TMSO)_2_C_6_H_4_CH_2_]^+^ 560 (10) [M + (TMS)_2_]^+^
		901 (45)	
**10**	431.3903 (100), [M − H]^−^	431 (100), [M − H]^−^	268 (100) [(TMSO)_2_C_6_H_4_CH_2_]^+^ 576 (20) [M + (TMS)_2_]^+^
	863.7925 (46), [2M − H]^−^	863 (46), [2M − H]^−^	
**11**	479.3919 (100), [M − H]^−^	479 (100), [M − H]^−^	268 (100) [(TMSO)_2_C_6_H_4_CH_2_]^+^ 624 (15) [M + (TMS)_2_]^+^
	343.3036 (51), [M − C_10_H_16_-H]^−^	959 (37), [2M − H]^−^	
**12**	459.4226 (100), [M − H]^−^	459 (100), [M − H]^−^	268 (100) [(TMSO)_2_C_6_H_4_CH_2_]^+^ 604 (20) [M-(TMS)_2_]^+^
	919.8549 (40), [2M − H]^−^	919 (40), [2M − H]^−^	

^a^ High-resolution mass spectrometry using a quadrupole-Time-of-Flight (QToF) tandem mass analyzer and electrospray ionization in negative ion mode coupled to an UFLC system; ^b^ Low-resolution mass spectrometry using a single quadrupole mass analyzer and electrospray ionization in negative ion mode and coupled to an UFLC system; ^c^ Electron-impact mass spectrometry using a quadrupole mass analyzer coupled to a GC system after derivatization with MSTFA.
